# Precision radiogenomics: fusion biopsies to target tumour habitats in vivo

**DOI:** 10.1038/s41416-021-01381-2

**Published:** 2021-04-16

**Authors:** Mireia Crispin-Ortuzar, Evis Sala

**Affiliations:** 1 Cancer Research UK Cambridge Centre, University of Cambridge, Cambridge, UK; 2 Department of Oncology, University of Cambridge, Cambridge, UK; 3 Department of Radiology, University of Cambridge, Cambridge, UK

**Keywords:** Ovarian cancer, Cancer imaging, Tumour heterogeneity

## Abstract

High-grade serous ovarian cancer lesions display a high degree of heterogeneity on CT scans. We have recently shown that regions with distinct imaging profiles can be accurately biopsied in vivo using a technique based on the fusion of CT and ultrasound scans.

## Main

In the last decade, the systematic, large-scale study of quantitative features extracted from routine medical images has grown exponentially due to increasing availability of larger datasets and more powerful computational resources. This new way of quantifying imaging data, known as radiomics, has been shown to provide valuable prognostic information across cancer types.^1,2^ One of the main challenges for radiomics studies is their interpretability; most of the time, the complexity of radiomics features is such that correlating them with specific visual patterns becomes almost impossible. As an alternative, integrative studies have used data-driven techniques to correlate radiomics features with different molecular or histopathological biomarkers in a more recent subfield, known as radiogenomics.^3^

Radiogenomics studies are crucial for the overall success of radiomics strategies. Not only are they essential to provide the biological underpinning that will enable the development of prospective clinical trials with radiomics biomarkers as end-points, but they provide the foundations for one of the biggest promises of the field—virtual biopsies. The idea of the virtual biopsy is that if the correlation between a given radiomic signature and its underlying molecular profile is sufficiently reliable, the signature itself can be used as a surrogate, potentially obviating the need for invasive tissue sampling procedures.^4^ This ability to strategically decide whether taking a tissue biopsy is necessary or not could be transformative for some of the most complex and aggressive types of cancer, where radiomics features display striking heterogeneity even within a single lesion, defining so-called “habitats”.^5^ For example, in high-grade serous ovarian cancer (HGSOC), our previous work shows that patients with higher degrees of heterogeneity between habitats on CT scans have significantly worse prognosis.^6^

Habitat-based heterogeneity in HGSOC lesions has been shown to capture underlying differences in phylogenetic evolution^7^ and T-cell infiltration^8^ in several pioneering studies, but the difficulty of the experiments has significantly limited the number of patients included. Crucially, these studies have so far focused on tissue obtained from surgical tissue samples, which are easier to obtain as the resected specimen is directly accessible. Even then, the experiments have required the development of new technologies based on 3D printing to enable accurate co-registration between habitats and tumour tissue.^9^ The challenge of guiding biopsies to habitats of interest in vivo, which is critical to understand the evolution of the tumour microenvironment before and during therapy, had so far been unexplored. One key obstacle is that ultrasound guidance is the most common form of image-guided biopsy, while radiomics habitats are typically defined on CT or MRI scans.

**Fig. 1:**
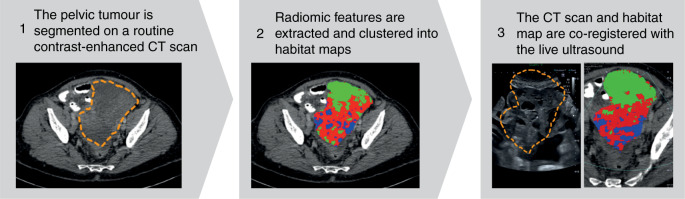
Schematic of the procedure used to obtain habitat-guided fusion biopsies. The steps are, from left to right: (1) segmentation of the lesion on a CT scan (dashed line); (2) extraction of habitat maps, here represented by three different colours; and (3) real-time CT–ultrasound co-registration. Figure reproduced from ^10^ under a Creative Commons Attribution 4.0 International License (http://creativecommons.org/licenses/by/4.0/).

We recently developed a study with the specific aim of demonstrating the feasibility of a new technique to sample CT-based habitats in vivo using ultrasound guidance in HGSOC patients.^10^ The technology relies on the live co-registration of a previously acquired CT scan and the ultrasound being used to guide the biopsy. Once the co-registration has been achieved, the habitats computed on the coordinate system of the CT scan can be directly overlaid on top of the ultrasound image and used to precisely and strategically guide the needle (Fig. 1).

Two main questions needed to be addressed to confirm the feasibility of the technique; the integration into clinical pathways and the accuracy of the result. We demonstrated the procedure for six patients, obtaining biopsies from a pelvic lesion in two of them, and from an omental lesion in four. We were able to extract habitat maps for each of them using contrast-enhanced CT scans acquired for disease staging as part of the standard of care. The CT–ultrasound fusion was performed using the clinically approved and commercially available US system from Canon Medical Systems; as a result, the standard clinical biopsy protocols were followed, with the only difference that the system was targeted to a habitat within the lesion rather than just the lesion. The procedure has now been integrated into our standard-of-care pathway for US-guided biopsies in ovarian cancer, adding up to 15 minutes to the standard biopsy procedure time. The segmentation and habitat computation are largely automated and do not add any extra time for the patient. The fusion system relies on a landmark-based system, and we were able to achieve co-registration quality indicators of 8/10 to 10/10 in all six cases. Ultrasound-guided biopsies in patients with suspected ovarian carcinoma have been shown to be safe;^11^ we did not observe any adverse events in this cohort.

To evaluate the accuracy of the co-registration for the tumour specifically, we assessed the overlap between the manually-segmented contours of the lesions as seen on ultrasound and CT. The average Dice similarity coefficient (DSC) of the pelvic lesions was 0.78, which indicated a high co-registration accuracy and therefore the adequacy of the method. Omental lesions, on the other hand, had an average DSC of 0.36. Several factors may have contributed to this lower accuracy: the omental deposits had a smaller volume and had more flexible positions compared to the larger, more fixed pelvic lesions. In addition, the anterior-posterior axis has a higher degree of misregistration due to the local pressure of the ultrasound probe. These results were encouraging and suggest that with some improvements—such as patient tracker systems that correct for local movements—the technique will be able to provide accurate habitat guidance not only for pelvic lesions but also for the more challenging omental metastases.

The ability to obtain habitat-guided biopsies routinely in HGSOC patients has the potential to significantly impact the way we understand and manage the disease. In general terms, guiding the biopsies to specific habitats will allow us to maximise the usefulness of the information obtained; in the long term, it may even be possible to rely on entirely virtual, imaging-based biopsies in some cases. Crucially, being able to track the evolution of the heterogeneous composition of the disease via the combination of habitat imaging and habitat-guided biopsies will make it possible to assess response more precisely—which is vital for both the drug development process and for clinical decision-making and treatment adaptation. Our study bridges one of the key technical barriers necessary to achieve this vision.

## Data Availability

Not applicable.
